# GPI-anchored glutathione S-transferase as marker allows affinity sorting of transfection-positive cells

**DOI:** 10.3389/fmolb.2022.1016090

**Published:** 2022-09-29

**Authors:** Shumin Ma, Lele Yang, Qingqing Zuo, Qilai Huang

**Affiliations:** Shandong Provincial Key Laboratory of Animal Cell and Developmental Biology, School of Life Science, Shandong University, Qingdao, China

**Keywords:** cell transfection, glutathione S-transferase, glutathione, GPI, cell sorting

## Abstract

Cell transfection efficiency is still a limiting factor in gene function research. A method that allows isolation and enrichment of the transfection-positive cells is an effective solution. Here, we report a transfection-positive cell sorting system that utilizes GPI-anchored GST (Glutathione S-transferase) as a plasmid marker. The Glutathione S-transferase fusion protein will be expressed and displayed on the cell surface through GPI anchor, and hence permits the positive cells to be isolated using Glutathione (GSH) Magnetic Beads. We prove that the system works efficiently in both the adherent Lenti-X 293T cells and the suspension K-562 cells. The affinity cell sorting procedure efficiently enriched positive cells from 20% to 98% in K-562 cells. The applications in gene knockdown and overexpression experiments in K-562 cells dramatically enhanced the extent of gene alteration, with the gene knockdown efficiency increasing from 7% to 60% and the gene overexpression level rising from 47 to 253 times. This Glutathione S-transferase affinity transfection-positive cell sorting method is simple and fast to operate, large-instrument free, low cost, and hence possesses great potential in gene function study *in vitro*.

## Introduction

Cell transfection is a powerful tool for *in vitro* gene function and regulation study (Hahn and Scanlan, 2010; Kim and Eberwine, 2010; Stepanenko and Heng, 2017). However, the transfection efficiency is still low for most primary cells and suspension cells, especially for large-size plasmids, and it is still hard to substantially improve the transfection efficiency (Kim and Eberwine, 2010). It severely limits the gene function study at the cellular level (Hu et al., 2016; Zhang et al., 2019). Although delivering a gene with a viral vector can alleviate the situation to some extent, it will bring extra stress on the cells, interact with endogenous cellular signal transduction components and cause cytotoxicity (Chen et al., 1999; Venticinque and Meruelo, 2010; Wu et al., 2021). In addition, the virus packaging process will take extra time.

Besides trying to improve cell delivery efficiency, it is an optimal strategy to develop a method that can sort and enriches transfection-positive cells. The existing methods for phenotypic selection of genetically modified mammalian cells are mainly based on three types of phenotypic markers: an exogenous drug resistance gene that permits screening for positive cells, a fluorescent protein, such as GFP, that allows isolating positive cells by FACS (fluorescence-activated cell sorting) (Chen et al., 1999), or a membrane protein that enables antibody-based immunomagnetic selection of positive cells (Plouffe et al., 2015). Even though commonly used in gene function study, all these methods have limitations in application. For the antibiotics screening method, due to the differential drug sensitivity of each cell line, it is usually necessary to conduct a pre-experiment to determine the working drug concentration before the formal experiment. It makes experiments complex, time-consuming and labor-intensive. On the other hand, the puromycin resistance gene expression or drug treatment itself may bring about unknown side effects on gene function (Moran et al., 2009). Although the FACS method saves time and labor, the instrument is usually not readily available to common laboratories because of its expensive cost. In addition, the strong laser used to excite fluorescence may also damage cells during the sorting process (Ren et al., 2019). Besides, even though the cell sorting speed has achieved great progress, it still limits the application in experiments requiring a vast number of positive cells (Arnold and Lannigan, 2010; Ren et al., 2015). The immunomagnetic positive cell selection method relies on antibodies targeting the overexpressed exogenous protein. Besides having the high-cost issue, the antibody might also interact with the endogenous proteins and cause non-specificity. (Wei et al., 2001; Bernard et al., 2002; Mahmoudi et al., 2012).

Recently, we developed a gene transfer-positive cell sorting system using membrane anchoring Twin-Strep-Tag (Yang et al., 2022). The system allows efficient enrichment of gene transfection-positive cells and can boost gene functional studies, including gene overexpression, gene knockdown and gene editing experiments et al. However, the cell sorting system utilizes Strep-Tactin magnetic beads to bind and separate positive cells, which still has the space to reduce cost.

Glutathione-S transferase (GST) is a multifunction enzyme that catalyzes the conjugation of glutathione to electrophilic compounds (Salinas and Wong, 1999). It has been popularly used as an affinity tag in the Glutathione-based affinity purification of fusion proteins or in the pull-down assay investigating protein-protein interaction (Schafer et al., 2015; Kim and Hakoshima, 2019). In this study, we use GST as the affinity tag to construct a new transfection-positive cell sorting system. The transfection-positive cells will express GPI-anchoring GST and can be bound and isolated using GSH magnetic beads, which have a lower price than Strep-Tactin magnetic beads. The system permits efficient positive-cell sorting in both suspension K-562 and adherent Lenti-X 293T cells and greatly promotes gene functional studies of gene knockdown and overexpression experiments.

## Results

### Design the membrane anchoring glutathione S-transferase fusion maker

**FIGURE 1:**
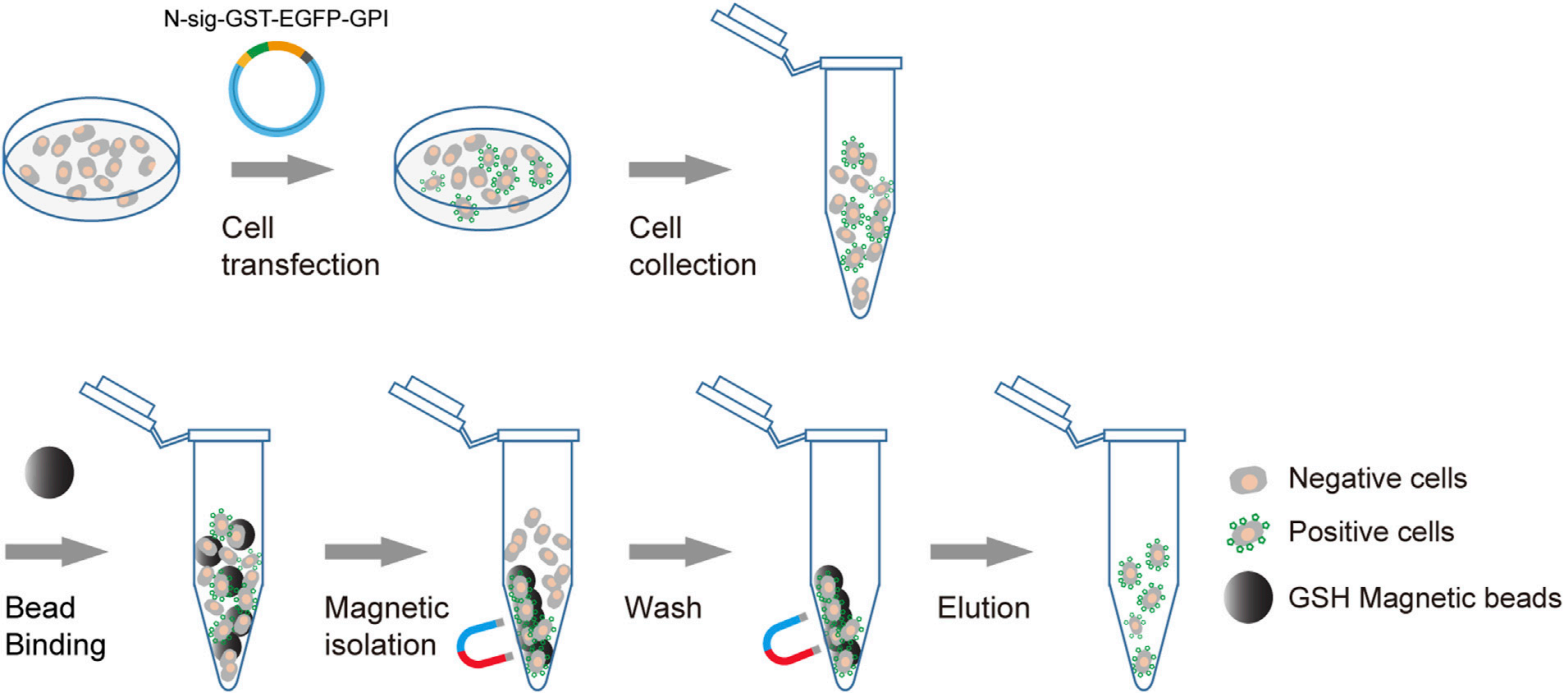
Schematic diagram of affinity cell sorting. The plasmids encoding the membrane-anchored GST-EGFP tag will be transfected into target cells. The positive cells will express the GST-EGFP tag on the cell surface and hence can be enriched through affinity cell sorting using GSH magnetic beads. The separation of the bead/cell complex can be performed by staying on a magnetic stand or by free settling. The transfection-positive cells are highlighted with green markers on the cell surface.

We use membrane anchoring GST molecule as the affinity tag to allow the transfection-positive cells to be isolated using GSH beads (Figure 1). Furthermore, to enable the positive cells to be conveniently monitored by fluorescence microscopy or flow cytometry, we introduce an in-frame EGFP to the C terminal of GST. To obtain efficient membrane translocation, we tested six membrane anchoring modules for their ability to translocate the GST fusion to the cell surface. Three modules are GPI-anchored protein signal sequences from DAF, BY55, and CEAM7, and the other three are transmembrane domains (TMDs) from ITAV, ITA5, and ITB3 as used previously (Yang et al., 2022).

### GPI anchoring modules transport Glutathione S-transferase to the cell membrane efficiently

**FIGURE 2:**
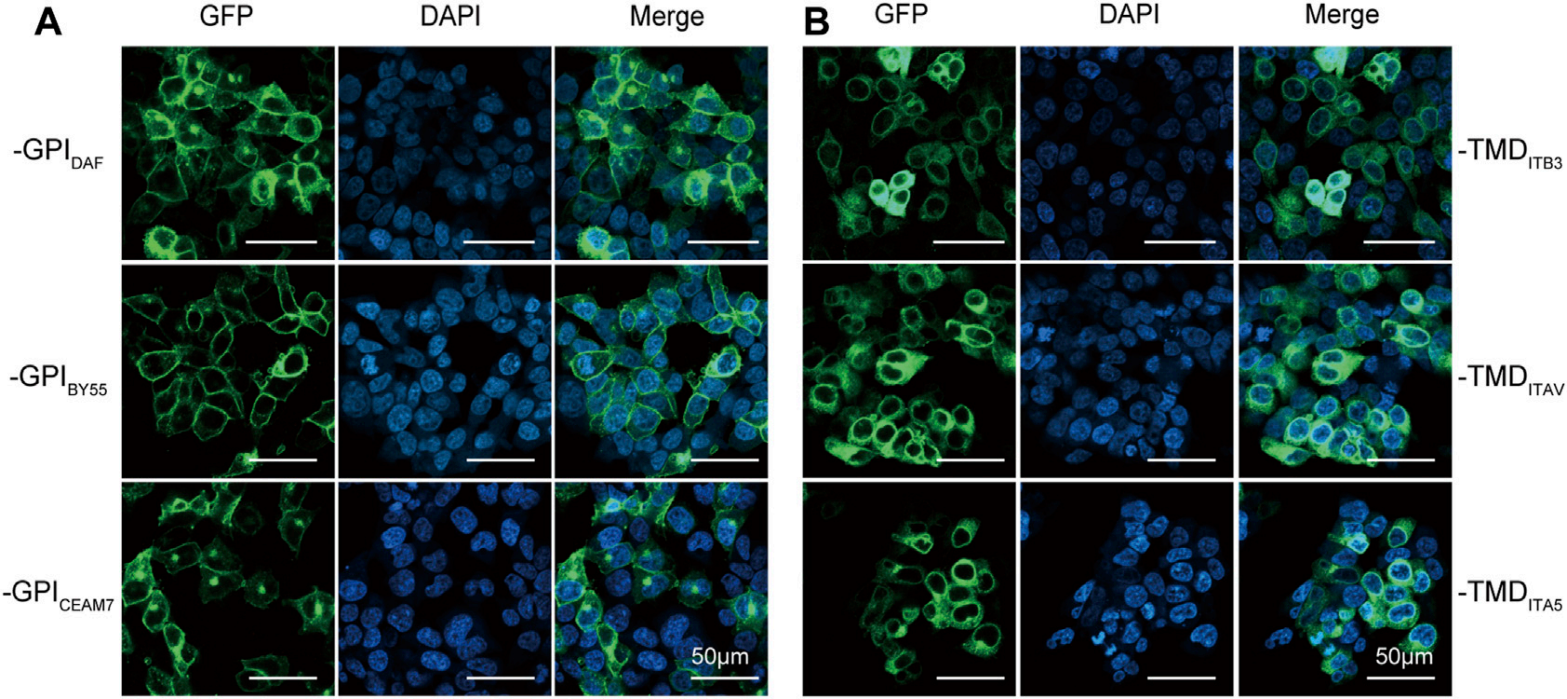
Membrane translocating of six GST-EGFP variants. Confocal fluorescence microscopy analysis of Lenti-X 293T cells transfected with plasmids encoding the GPI-type variants pEGFP-GST-GPI_DAF_, pEGFP-GST-GPI_BY55_, and pEGFP-GST-GPI_CEAM7_ **(A)** and TMD-type variants pEGFP-GST-TMD_ITB3_, pEGFP-GST-TMD_ITA5_ and pEGFP-GST-TMD_ITAV_ **(B)**. The nucleus was stained with DAPI (4′,6-diamidino-2-phenylindole). Cells were observed at ×200 magnification, and the scale bar represents 50 µm.

We transfected Lenti-X 293T cells with plasmids expressing each GST tag to compare their ability in membrane translocation. The confocal fluorescence microscopy analysis showed that the three GPI type tags, GST-EGFP-GPI_DAF_, GST-EGFP-GPI_BY55,_ and GST-EGFP-GPI_CEAM7,_ were efficiently expressed and transported to the cell membrane (Figure 2A). However, the three TMD type tags, GST-EGFP-TMD_ITB3_, GST-EGFP-TMD_ITAV,_ and GST-EGFP-TMD_ITA5_, remained mainly in the cytoplasm (Figure 2B). It indicates that the full function of membrane targeting might require extra amino acids outside the minimal transmembrane domain.

To investigate whether the ectopic expression of the GPI type sorting tags alters cell physiology, we transfected the sorting plasmids into K-562 cells and performed cell proliferation assay. The results showed that the cells expressing GST-EGFP-GPI_DAF_, GST-EGFP-GPI_BY55,_ and GST-EGFP-GPI_CEAM7_ exhibited comparable proliferation ability to cells expressing the single EGFP only (Supplementary Figure S1).

### Affinity cell sorting enriches transfection-positive cells

**FIGURE 3:**
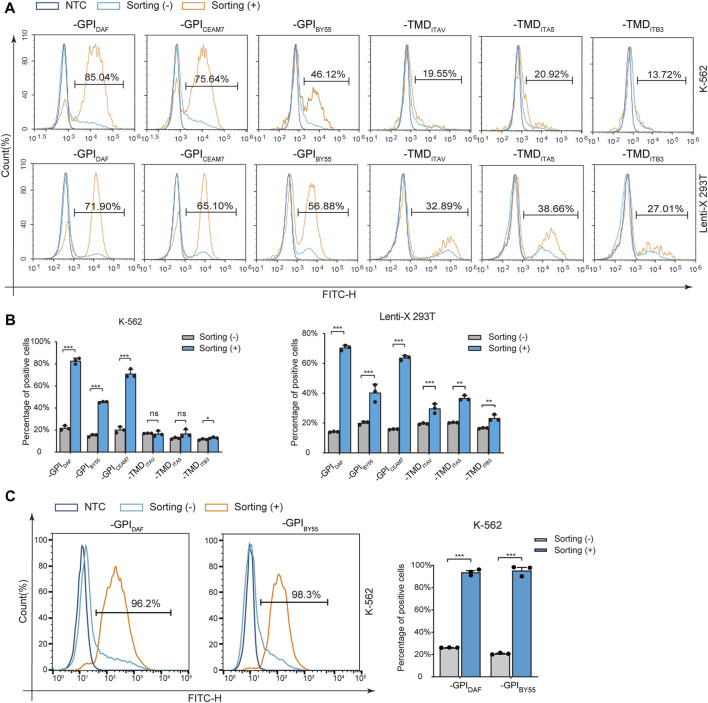
Positive-cell enrichment fold assay with Flow cytometry. **(A)** Representative flow cytometry histograms of K-562 and Lenti-X 293T cells transfected with six sorting tag plasmids, with or without cell sorting. The dark blue layer represents NTC cells without transfection, and the light blue layer and the orange layer represent transfected cells without (−) or with cell sorting (+). The value in the region gate represents the percentage of positive cells in the enriched cells. **(B)** The bar chart shows the positive cell percentage value from the above flow cytometry analysis. **(C)** Flow cytometry analysis of K-562 cells transfected with plasmids expressing GST-EGFP-GPI_DAF_ and GST-EGFP-GPI_BY55_ correspondingly and enriched through affinity cell sorting of free settling strategy. The layers represent cells without sorting (-) (light blue), sorted (+) (orange), and negative control (NTC, dark blue). The bar chart on the right is the quantitative positive percentage value from the left flow cytometry analysis. Values are from three biological replicates. Means ± SD, ns stands for no significance, *p < 0.05, **p < 0.01, ***p < 0.001, two-tailed Student’s t-test.

Then we transfected Lenti-X 293T and K-562 cells with the six variants separately and performed affinity cell isolation using GSH magnetic beads 30 h post-transfection. The enriched cells were directly analyzed by flow cytometry to determine the positive cell ratio or subjected to RNA preparation and RT-qPCR analysis to determine the enrichment fold with GST RNA expression. The flow cytometry analysis showed that affinity cell sorting with GST-EGFP-GPI_DAF_, GST-EGFP-GPI_BY55,_ and GST-EGFP-GPI_CEAM7_ significantly increased the EGFP positive cell ratio in both Lenti-X 293T and K-562 cells (Figures 3A,B). Specifically, cell sorting of GST-EGFP-GPI_DAF_ dramatically increased the positive ratio from 20% to 81% in K-562 cells and from 14% to 71% in Lenti-X 293T cells. Cell sorting of GST-EGFP-GPI_BY55_ increased the positive ratio from 15% to 46% in K-562 cells and from 20% to 40% in Lenti-X 293T cells. For GST-EGFP-GPI_CEAM7,_ the positive ratio was increased from 20% to 75% in K-562 and from 18% to 65% in Lenti-X 293T cells (Figure 3B).

However, for the three TMD type markers, GST-EGFP-TMD_ITB3_, GST-EGFP-TMD_ITAV,_ and GST-EGFP-TMD_ITA5_, cell sorting did not significantly increase the positive cell ratio in K-562 cells and only function slightly in Lenti-X 293T cells (Figures 3A,B). Interestingly, when we optimized the cell sorting procedure by using free settling to replace magnetic separation, the EGFP positive cell percentage could be elevated from 20% to over 95% in K-562 cells for both GST-EGFP-GPI_DAF_ and GST-EGFP-GPI_BY55_ (Figure 3C).

**FIGURE 4:**
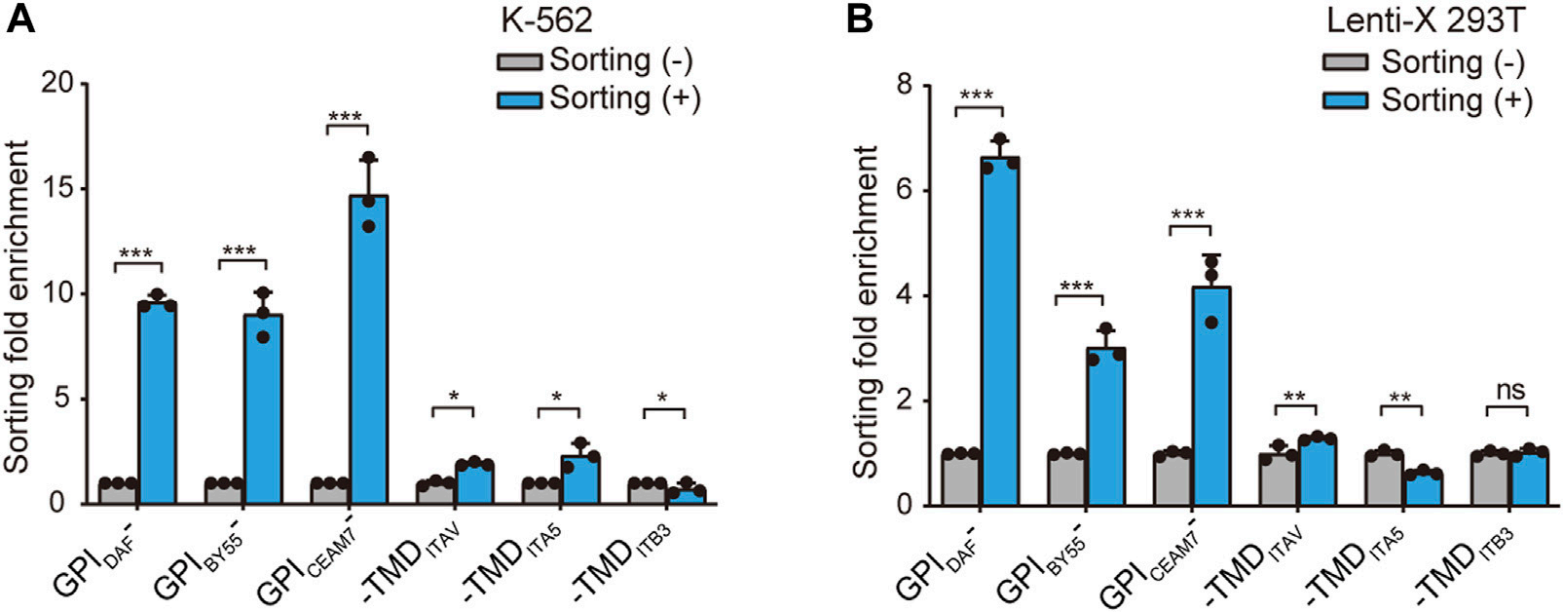
Positive-cell enrichment fold assay through GST RNA level. The bar chart shows the cell sorting fold enrichment of the six sorting tags in K-562 **(A)** and Lenti-X 293T cells **(B)**, determined by RT-qPCR analysis of GST RNA expression level. The affinity cell sorting was performed 30 h post-transfection. RNA was extracted, and the *GST* mRNA level relative to β-actin was determined. Values are from three biological replicates. Means ± SD, *p < 0.05, **p < 0.01, ***p < 0.001, two-tailed Student’s t-test.

Further, we prepared RNA from the sorted cells of the six GST makers and determined the GST mRNA level using the RT-qPCR method to calculate the enrichment fold. The results showed that GST-EGFP-GPI_DAF_ and GST-EGFP-GPI_CEAM7_ exhibited the highest enrichment fold in both K-562 and Lenti-X 293T cells (Figures 4A,B). Notably, in the sorted K-562 cells with the GST-EGFP-GPI_DAF_ marker, the GST expression level was 9.4 times that in the transfected cells before sorting. For GST-EGFP-GPI_CEAM7_, the sorted K-562 cells exhibited a GST expression level of 15 times the before sorting cells. (Figure 4A). In the sorted Lenti-X 293T cells with GST-EGFP-GPI_DAF_ and GST-EGFP-GPI_CEAM7_, the GST displayed expression levels of 6.6 times and 4.3 times to the before sorting cells, respectively (Figure 4B). Given their higher positive cell enrichment fold, we chose GST-EGFP-GPI_DAF_ or GST-EGFP-GPI_CEAM7_ to investigate their potential further to enrich positive cells in subsequent gene function studies.

### Sorted cells exhibit increased gene knockdown efficiency

**FIGURE 5:**
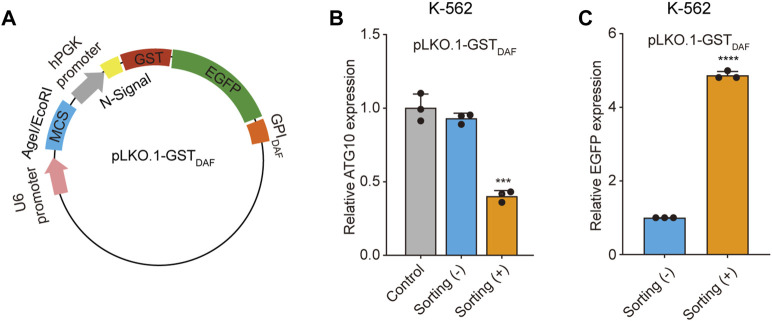
Sorted cells exhibit increased shRNA knockdown efficiency. **(A)** Schematic diagram of the plasmid encoding the GST-EGFP-GPI_DAF_ sorting tag. **(B)** The bar chart shows the relative *ATG10* gene expression level in K-562 cells transfected with the blank pLKO.1-GST_DAF_ vector (control), or vector expressing *ATG10* shRNA, with or without cell sorting. **(C)**The relative EGFP expression level in the above K-562 cells as determined by RT-qPCR analysis. The EGFP expression level was normalized with β-actin level. Values are three technical replicates. Means ± SD, *p < 0.05, **p < 0.01, ***p < 0.001, two-tailed Student’s t-test.

We first applied the cell sorting system in the shRNA gene knockdown experiment. To construct the shRNA knockdown vector pLKO.1-GST_DAF_, we inserted the GST-EGFP-GPI_DAF_ coding region into the pLKO.1 puro plasmid in place of the puromycin resistance gene. (Figure 5A). Then we designed shRNA targeting the *ATG10* gene, an E2-like enzyme involved in autophagy that catalyzes the conjugation of *ATG12* to *ATG5* (Kaiser et al., 2012; Kaiser et al., 2013). We transfected K-562 cells with the shRNA plasmid and performed affinity cell sorting using GSH magnetic beads to enrich the positive cells. The RT-qPCR analysis of *ATG10* expression level revealed that the affinity cell sorting led to a significantly increased shRNA knockdown efficiency of 60% in the sorted cells, compared to a knockdown efficiency of 7% in the before sorting cells (Figure 5B). Meanwhile, the EGFP expression level in the sorted cells exhibited an enrichment fold of 5 times compared to the before sorting cells (Figure 5C). It indicates that the positive cell sorting operation was efficient and had great potential to promote the apparent shRNA knockdown efficiency dramatically.

### Sorted cells display elevated gene overexpression level

**FIGURE 6:**
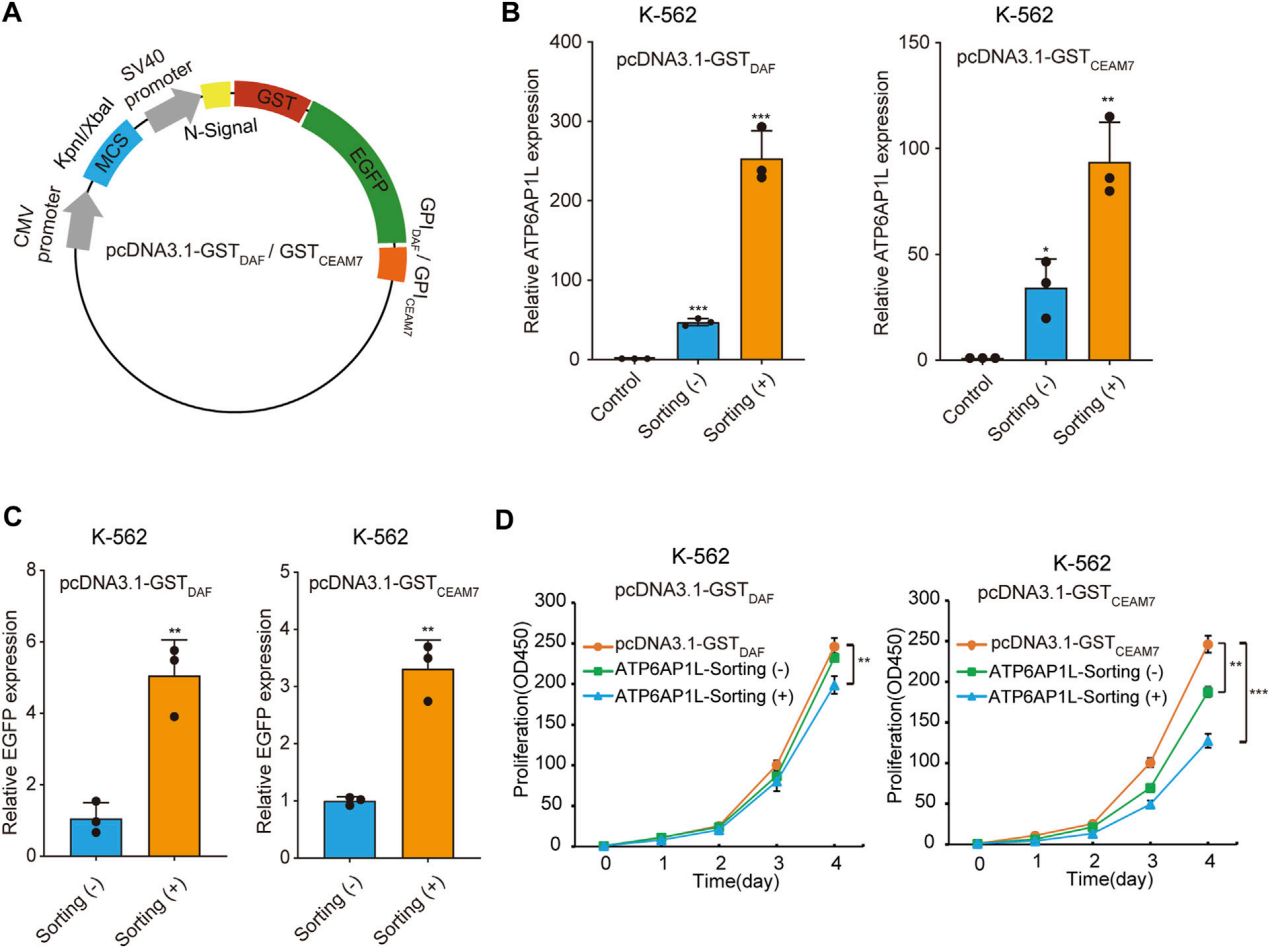
Sorted cells allow dramatically increased ectopic gene expression. **(A)** Schematic diagram of plasmids expressing GST-EGFP-GPI_DAF_ or GST-EGFP-GPI_CEAM7_ sorting tag. **(B)** The bar chart represents the *ATPAP1L* mRNA expression level in K-562 cells transfected with the blank vector (control) or the corresponding overexpression plasmid, determined by RT-qPCR. **(C)** The relative EGFP expression level in the above K-562 cells as determined by RT-qPCR analysis. The EGFP expression level was normalized with the β-actin level. **(D)** CCK-8 cell proliferation assay of K-562 cells transfected with *ATP6AP1L* expression plasmid, with or without affinity cell sorting. Cells transfected with the blank pcDNA3.1-GST_DAF_ or pcDNA3.1-GST_CEAM7_ plasmid were used as the negative control. Values are from three biological replicate wells. Means ± SD, *p < 0.05, **p < 0.01, ***p < 0.001, two-tailed Student’s t-test.

We further demonstrated the cell sorting strategy in a gene overexpression experiment. To construct the blank vectors for gene overexpression capable of affinity cell sorting, we inserted the coding sequence of GST-EGFP-GPI_DAF_ or GST-EGFP-GPI_CEAM7_ marker into pcDNA3.1 in place of the neomycin resistance gene and obtained pcDNA3.1-GST_DAF_ and pcDNA3.1-GST_CEAM7_ (Figure 6A). Then, we cloned the *ATP6AP1L* (ATPase H + Transporting Accessory Protein 1 Like), a gene mediating breast cancer predisposition of rs10514231 (Ma et al., 2021), into the two vectors under CMV promoter/enhancer and transfected K-562 cells. The RT-qPCR results showed that cell sorting operation dramatically increased the apparent expression level of the *ATP6AP1L* gene in the enriched cells with both expression plasmids (Figure 6B). Specifically, the pcDNA3.1-GST_DAF_-ATP6AP1L transfection resulted in an *ATP6AP1L* gene expression level of 47 times that of the control, and the affinity cell sorting procedure significantly elevated the apparent expression level in the enriched cells by 253 times that of the control. Similarly, transfection with pcDNA3.1-GST_CEAM7_-ATP6AP1L resulted in an mRNA level of 34 times that of the control, and affinity cell sorting promoted the expression level to 94 times that of the control. In addition, the EGFP expression level determined by RT-qPCR also confirmed the enrichment efficiency of the cell sorting operation, with five times for pcDNA3.1-GST_DAF_-ATP6AP1L and 3.3 times for pcDNA3.1-GST_CEAM7_-ATP6AP1L (Figure 6C).

### Cell sorting facilitates gene function study

The *ATP6AP1L* gene has been reported to be associated with proctitis (Pathak et al., 2020). We have recently found that *ATP6AP1L* overexpression in breast cancer cells suppressed cell proliferation (Ma et al., 2021). To explore whether cell sorting operation can highlight the biological function of the target gene, we performed a cell proliferation assay with K-562 cells transfected with the plasmid encoding the *ATP6AP1L* gene, with or without enrichment by affinity cell sorting. The results showed that the overexpression of *ATP6AP1L* gene with pcDNA3.1-GST_DAF_-ATP6AP1L and pcDNA3.1-GST_CEAM7_-ATP6AP1L could significantly inhibit the proliferation of K-562 cells compared with the control cells transfected with empty vectors, pcDNA3.1-GST_DAF,_ and pcDNA3.1-GST_CEAM7_, respectively. Notably, a more profound inhibitory effect was observed in the enriched cells with both expression vectors (Figure 6D). These results indicate that the affinity sorting system can increase gene modification to a larger extent and allow the observation of more profound gene biological functions.

## Discussion

Here we develop a transfection-positive cell sorting system utilizing GPI-anchored GST as the affinity tag, which enables the positive cells to be isolated using GSH magnetic beads. The affinity tag can successfully express in the positive cells and translocate to the external leaflet of the cell membrane. The in-frame EGFP module at the C-terminal of the GST tag allows the positive cells to be observed under fluorescent microscopy and quantified with flow cytometry. We prove that the sorting system works efficiently in both the adherent Lenti-X 293T and suspension K-562 cells to enrich positive cells to an extent comparable to or greater than the present FACS and MACS methods (Rubio et al., 2016; Pan and Wan, 2020). Furthermore, the cell sorting system can dramatically increase the apparent gene alteration fold, including shRNA knockdown and gene overexpression, and permits the observation of more profound gene biological function.

Our cell sorting system has several advantages compared to the existing transfection-positive cell sorting methods, including FACS, drug screening, and MACS. Compared to FACS, our GST affinity cell sorting system does not require expensive equipment and is less likely to cause mechanical cell damage (Dainiak et al., 2007). Notably, the cell sorting throughput is easily scalable for our method by simply scaling up or paralleled operation, whereas the FACS method is limited by the sorting speed of the cell sorter. Compared to the drug screening methods, our sorting system has a better generality with cell types and is much more time-saving. We do not require pre-experiment to determine the operating parameter for each cell line. In contrast, it is often obligatory for drug screening methods to clarify the case-dependent working concentration before the formal experiments, and the process of killing the transfection-negative cells usually takes several days. Importantly, our affinity cell sorting method does not cause cytotoxicity like the drug screening. For comparison with the MACS methods, the GST affinity cell sorting method is antibody free and hence has a low cost. Meanwhile, the EGFP module in our sorting tag allows convenient evaluation of the positive cells through fluorescent microscopy and flow cytometry. One antibody-free magnetic cell sorting method that displays a Streptavidin Binding Peptide (SBP) at the cell surface by the truncated Low Affinity Nerve Growth Receptor (LNGFRF) has been reported (Matheson et al., 2014). Given that the overexpression of LNGFR may promote osteogenic differentiation in rat ectomesenchymal stem cells (Li et al., 2017), the application of this cell sorting system may alter the cell growth state and bring about side effects on the study.

The recently reported positive cell sorting system utilizes GPI-anchored Twin-Strep-Tag as the affinity tag and allows the positive cells to be enriched with Strep-Tactin magnetic beads (Yang et al., 2022). It works efficiently in gene function studies, but however, the usage of Strep-Tactin magnetic beads still remains a big cost. As a comparison, the GPI-anchored GST tag cell sorting system has an obviously lower cost and meanwhile a comparable performance. For affinity sorting systems, the magnetic beads coupled with corresponding ligands constitute the main cost. According to the market price from Beaver Biomedical Engineering Co., Ltd., the price of GSH magnetic beads is less than one-third that of Strep-Tactin magnetic beads.

Reduced GSH is widely expressed in plant, animal, and fungal cells (Wu et al., 2004). The purification of GST fusions from mammalian cells using GSH-coupled magnetic beads usually requires the removal of the competitively binding endogenous GSH (Tessema et al., 2006). However, the GPI-anchoring module allows the GST fusion to express in the endoplasmic reticulum (ER) lumen and translocate to the out leaflet of the cell membrane through the Golgi apparatus (Kinoshita, 2020), making GST free from the endogenous GSH ligands. It allows GPI-GST-based cell sorting to be performed directly without the requirement to remove competitive intracellular GSH. However, researchers need to pay attention to whether the cell culture medium itself contains GSH ingredients. For example, RPMI-1640 contains 1.0 mg/L of reduced GSH, and McCoy’s 5A Modified Medium contains 0.5 mg/L. On the contrary, the common Dulbecco’s Modified Eagle’s Medium (DMEM), Nutrient Mixture, Ham’s F-12, DMEM/Ham’s Nutrient Mixture F-12 (50:50), and Iscove’s Modified Dulbecco’s Medium (IMDM) are free of GSH.

In brief, we develop a gene transfection-positive cell sorting system utilizing GPI-anchored GST as the affinity tag that enables positive cell isolation using GSH magnetic beads. The sorting system is time and cost-saving and widely applicable to both adherent cells and suspension cells. The EGFP module in the sorting tag can also be replaced by other fluorescent proteins such as YFP, mCherry, mStrawberry, mOrange et al. to make the sorting system more selective. Hence the sorting system in this paper holds great promise in gene function studies.

## Materials and methods

### Cell culture

The K-562 and Lenti-X 293T cells were purchased from the American Type Culture Collection (CCL-243, ATCC) and Clontech (#632180), respectively. They were maintained in IMDM medium (Invitrogen), and DMEM medium (Invitrogen) correspondingly, supplemented with 10% fetal bovine serum (FBS) (S711-001S, Lonsera) and 1% Penicillin-Streptomycin (SV30010, HyClone). Both cells were cultured at 37°C with 5% CO_2_, generally subcultured every 2–3 days, and regularly tested for *mycoplasma* using mycoblue®*mycoplasma* Detector (D101-02, Vazyme).

### Sorting vector construction and gene cloning

The DNA sequences encoding the GST, the N-terminal signal peptides, and the C-terminal membrane-placing module of the six GPI/TMDs (Supplementary Table S1) were synthesized and supplied in pUC57a by GENEWIZ. The N-terminal signal peptides, GST coding sequence, and C-terminal membrane-placing modules were then PCR amplified separately from the synthesized genes. The EGFP coding sequence was amplified from the pEGFP-C2 plasmid. The PCR products were then joined through overlap extension PCR (SOE PCR) at an equal mole ratio to obtain the six types of N-signal-GST-EGFP-GPI/TMDs coding regions. Further, the SOE PCR products were cloned into FastDigest BshTI/FastDigest BglII (Thermo Fisher) linearized pEGFP-C2 vector using ClonExpress^®^ II One Step Cloning Kit (C112, Vazyme) to obtain the six sorting vectors, pEGFP-GST-GPI_DAF_, pEGFP-GST-GPI_BY55_, pEGFP-GST-GPI_CEAM7_, pEGFP-GST-TMD_ITB3_, pEGFP-GST-TMD_ITA5,_ and pEGFP-GST-TMD_ITAV_. All related primers are listed in Supplementary Table S2.

To construct the sorting vector pLKO.1-GST_DAF_ for shRNA gene knockdown experiment, we amplified the coding sequence of N-signal-GST-EGFP-GPI_DAF_ from the pEGFP-GST-GPI_DAF_ plasmid and joined it with FastDigest KpnI/FastDigest BamHI (Thermo Fisher) linearized pLKO.1 vector using ClonExpress^®^ II One Step Cloning Kit. The shRNA sequence (TRCN0000322995) targeting *ATG10* was designed according to MISSION^®^ shRNA Plasmid DNA (MERCK) and synthesized and joined the FastDigest BshTI/FastDigest EcoRI linearized (Thermo Fisher) pLKO.1-GST_DAF_ vector with T4 DNA ligase (EL0011, Thermo Fisher). Primer sequences are shown in Supplementary Table S2.

To construct the sorting vector for gene overexpression, we amplified the whole expression cassette of N-signal-GST-EGFP-GPI_DAF_ and N-signal-GST-EGFP-GPI_CEAM7_ from pEGFP-GST-GPI_DAF_ and pEGFP-GST-GPI_CEAM7_ vectors and joined them with PCR linearized pcDNA3.1 vector using ClonExpress^®^ II One Step Cloning Kit. The resulted plasmids are pcDNA3.1-GST_DAF_ and pcDNA3.1-GST_CEAM7_. Subsequently, the *ATP6AP1L* coding sequence was amplified from the cDNA of T47D cells and inserted into the FastDigest KpnI/FastDigest XbaI (Thermo Fisher) linearized pcDNA3.1-GST_DAF_ and pcDNA3.1-GST_CEAM7_ vectors using T4 DNA ligase (EL0011, Thermo Fisher). The related primer sequences are shown in Supplementary Table S2.

### Cell transfection

The endotoxin-free plasmids were prepared with plasmid miniprep plus purification kit (DP01-Plus-300, GeneMark), purified by ethanol precipitation, and subjected to cell transfection. K-562 cells were transfected using the Lipofectamine 2000 Reagent (11,668–019, Invitrogen) following the manufacturer’s instructions. Briefly, cells were seeded in the 6-well plate with 3 × 10^5^ cells per well and transfected with 2 μg plasmids using 6 μL Lipofectamine 2000 regent. DNA and transfection reagent was diluted in 250 μL Opti-MEM (Gibco) separately, mixed and incubated for 10 min at room temperature. The DNA-lipofectamine mixtures were applied to cells gently, and the cells were put back in the 37°C incubator for 24–48 h.

For transfection of Lenti-X 293T, cells were seeded in the 6-well plate on the first day and transfected on the second day when the cells reached approximately 70%–90% confluent, as described previously (Ren et al., 2021). Briefly, 1.5 μg plasmids DNA and 3 μL of 1 mg/ml Polyethylenimine (PEI, 408,727-sigma) transfection reagent were diluted in 250 μL of FBS-free DMEM (Invitrogen) separately. They were mixed thoroughly and incubated for 10 min at room temperature. Finally, the cells were exchanged with pre-warmed FBS-free DMEM medium, and then the DNA-PEI mixture was added to the cells gently. The medium was replaced with fresh complete DMEM medium 6–8 h after transfection, and cells were grown for 24–48 h.

### Confocal fluorescence microscopy analysis

Lenti-X 293T cells were seeded on cell slides in 24 well-plate on the first day and transfected on the next day when cells reached 30%–50% confluence. Briefly, 1.5 μg of pEGFP-GST-GPI_BY55_, pEGFP-GST-GPI_DAF_, pEGFP-GST-GPI_CEAM7_, pEGFP-GST-TM_ITB3_, pEGFP-GST-TM_ITA5_ and pEGFP-GST-TM_ITAV_ plasmids were transfected using 3 μL of 1 mg/ml PEI as described above. 48 h after transfection, cells were washed twice with pre-warmed PBS and fixed with 4% paraformaldehyde (PFA) for 10–15 min. After twice washing with PBS, the nuclei were counterstained with 10 μg/ml DAPI (4′,6-diamidino-2-phenylindole) (C0060, Solarbio) for 10 min at 37°C. Finally, the slides were fixed with Antifade Mounting Medium (S2100, Solarbio), and images were acquired using a confocal laser scanning microscope (LSM900, ZEISS).

### Affinity cell sorting

Affinity cell sorting was performed 30 h post-transfection. For each sorting reaction, 150 μL GSH magnetic beads (70,601–100, Beaverbio) were prepared by twice washing with 1 ml PBS and resuspended in 500 μL binding buffer (DMEM with 2% FBS). For cell sorting of Lenti-X 293T, cells were rinsed twice with 500 μL PBS and then treated with 300 μL Non-enzymatic Cell Dissociation Solution (13151014, Gibco) for 4–6 min at 37°C to detach cells. The dissociation process was terminated with fresh complete DMEM medium. The cells were collected, washed twice with 500 μL PBS, and resuspended in 500 μL binding buffer (DMEM with 2% FBS). As to the K-562 cells, cells were collected directly, washed twice with 500 μL 1 × PBS, and resuspended in 500 μL binding buffer.

One-fifth of the cell suspension was reserved as the without-sorting control sample. The left cells were gently mixed with the prepared GSH magnetic beads suspension and incubated on a rotator at 10 rpm for 15 min at room temperature. Then the tubes were placed on the magnetic stand for 1 min to separate the bead/cell complexes. The supernatant was removed carefully, and the bead/cell complexes were washed once with washing buffer (DMEM with 2% FBS). Finally, the transfect-positive cells were eluted with 300 μL DMEM on a rotator with 15 rpm for 5 min at room temperature. The cells were collected and subjected to flow cytometry analysis or RNA extraction.

Alternatively, we also optimized the cell sorting procedure by separating the bead/cell complexes through free settling instead of using the magnetic stand, as described previously (Yang et al., 2022). Briefly, D-PBS containing 0.1% BSA was used to prepare GSH magnetic beads and cell suspension, and the binding reaction was performed with a 1.5 ml volume in the1.5 ml tube. After the incubation step, the tube was put on a stand for 1 min. The beads settled faster than and hence separated from the unbound cells easily. The bead/cell complexes were resuspended in 1.5 ml D-PBS/BSA solution and performed free settling again to separate from the free cells. The transfection-positive cells were then released from the beads as described above.

### RNA extraction and RT-qPCR

Total RNA was extracted using the GeneJET RNA Purification Kit (K0732, Thermo) according to the manufacturer’s instructions. The RNA was then treated with RapidOut DNA Removal Kit (K2981, Thermo) according to the user guide to remove trace genomic DNA residue. Then cDNA was synthesized using High-Capacity cDNA Reverse Transcription Kits (4374967, Applied Biosystems) with the accompanied random primers, as described previously (Ren et al., 2021). The reverse transcription reaction was performed by incubation at 25°C for 10 min, followed by 120 min at 37°C, and heat inactivation at 85°C for 5 min. The cDNA products were used directly for subsequent experiments or stored in a −80°C refrigerator.

The mRNA expression levels of given genes in the transfected cells with or without cell sorting enrichment were determined by qPCR using Taq388 mix (Du et al., 2022) on a QIAGEN Q-Rex machine (Qiagen, Germany). The qPCR was performed with the following program: 95°C for 5 min of initial denaturation, then 40 cycles of 95°C for 30 s, 60°C for 30 s and 72°C for 10 s with fluorescence acquirement, followed by a final melting curve step. The relative expression of target genes was determined using the comparative Ct method and normalized with the endogenous ACTB (β-actin) gene. In the gene knockdown and overexpression experiments, the corresponding empty vectors were used as the negative control in the transfection. The statistical significance was calculated by a two-tailed Student’s t-test. All the related primers are listed in Supplementary Table S3.

### Flow cytometry analysis

The cells from the cell sorting procedure were directly subject to flow cytometry analysis using NovoCyte™ Flow Cytometer (ACEA Bio). The GFP signals were detected with the FITC channel, using a detector gain that positions the negative control cell peak around 1 × 10^2^ and 1 × 10^3^. At least 10,000 events were recorded for each sample with a low-speed flow rate.

### Cell viability and proliferation assays

The transfected K-562 cells with or without cell sorting and the negative control cells transfected with empty plasmids pcDNA3.1-GST_DAF_ or pcDNA3.1-GST_CEAM7_ were counted and seeded into 96-well plates in triplicates at a density of 1,000 per well. Cell viability and proliferation were determined using the CCK-8 kit (MA0218, Meilun) at 0, 24, 48, 72, and 96 h post seeding, as described previously (Yang et al., 2022). Briefly, 10 μL of CCK-8 reagent was added to the 100 μL cell suspension and incubated for 3 h at 37°C. Then the absorbance at 450 nm was measured with 600 nm as the reference wavelength. The statistical significance was calculated by a two-tailed Student’s *t*-test.

## Data Availability

The datasets presented in this study can be found in online repositories. The names of the repository/repositories and accession number(s) can be found in the article/Supplementary Material.
