# Supplementary material for: GPI-anchored glutathione S-transferase as marker allows affinity sorting of transfection-positive cells
Source: Front Mol Biosci. 2022 Sep 29;9:1016090. doi: 10.3389/fmolb.2022.1016090 (PMC9558730; doi:10.3389/fmolb.2022.1016090)
Supplement: Supplementary file 1 [file Table1.DOCX]

Supplementary Material

# Supplementary Figures


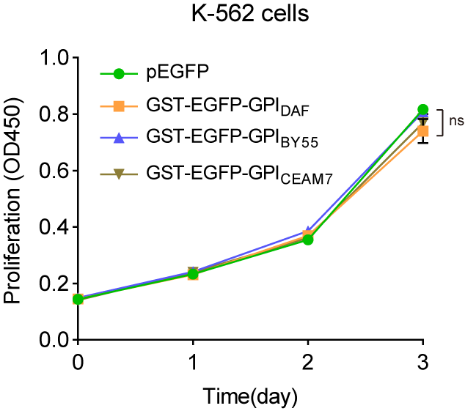


**Supplementary Figure 1. Cell proliferation assay of K-562 cells expressing three GPI-type soring tags.** CCK-8 cell proliferation assay of K-562 cells transfected with plasmids expressing EGFP, GST-EGFP-GPI_DAF_, GST-EGFP-GPI_BY55_, GST-EGFP-GPI_CEAM7_ separately. Values are from three biological replicate wells. Means ± SD, ns: no significant difference, two-tailed Student’s t-test.

# Supplementary Tables

**Supplementary Table 1**

DNA sequences of the six sorting tags

| GST^a^ | TCCCCTATACTAGGTTATTGGAAAATTAAGGGCCTTGTGCAACCCACTCGACTTCTTTTGGAATATCTTGAAGAAAAATATGAAGAGCATTTGTATGAGCGCGATGAAGGTGATAAATGGCGAAACAAAAAGTTTGAATTGGGTTTGGAGTTTCCCAATCTTCCTTATTATATTGATGGTGATGTTAAATTAACACAGTCTATGGCCATCATACGTTATATAGCTGACAAGCACAACATGTTGGGTGGTTGTCCAAAAGAGCGTGCAGAGATTTCAATGCTTGAAGGAGCGGTTTTGGATATTAGATACGGTGTTTCGAGAATTGCATATAGTAAAGACTTTGAAACTCTCAAAGTTGATTTTCTTAGCAAGCTACCTGAAATGCTGAAAATGTTCGAAGATCGTTTATGTCATAAAACATATTTAAATGGTGATCATGTAACCCATCCTGACTTCATGTTGTATGACGCTCTTGATGTTGTTTTATACATGGACCCAATGTGCCTGGATGCGTTCCCAAAATTAGTTTGTTTTAAAAAACGTATTGAAGCTATCCCACAAATTGATAAGTACTTGAAATCCAGCAAGTATATAGCATGGCCTTTGCAGGGCTGGCAAGCCACGTTTGGTGGTGGCGACCATCCTCCAAAA |
| --- | --- |
| EGFP^b^ | CCGGTCGCCACCATGGTGAGCAAGGGCGAGGAGCTGTTCACCGGGGTGGTGCCCATCCTGGTCGAGCTGGACGGCGACGTAAACGGCCACAAGTTCAGCGTGTCCGGCGAGGGCGAGGGCGATGCCACCTACGGCAAGCTGACCCTGAAGTTCATCTGCACCACCGGCAAGCTGCCCGTGCCCTGGCCCACCCTCGTGACCACCCTGACCTACGGCGTGCAGTGCTTCAGCCGCTACCCCGACCACATGAAGCAGCACGACTTCTTCAAGTCCGCCATGCCCGAAGGCTACGTCCAGGAGCGCACCATCTTCTTCAAGGACGACGGCAACTACAAGACCCGCGCCGAGGTGAAGTTCGAGGGCGACACCCTGGTGAACCGCATCGAGCTGAAGGGCATCGACTTCAAGGAGGACGGCAACATCCTGGGGCACAAGCTGGAGTACAACTACAACAGCCACAACGTCTATATCATGGCCGACAAGCAGAAGAACGGCATCAAGGTGAACTTCAAGATCCGCCACAACATCGAGGACGGCAGCGTGCAGCTCGCCGACCACTACCAGCAGAACACCCCCATCGGCGACGGCCCCGTGCTGCTGCCCGACAACCACTACCTGAGCACCCAGTCCGCCCTGAGCAAAGACCCCAACGAGAAGCGCGATCACATGGTCCTGCTGGAGTTCGTGACCGCCGCCGGGATCACTCTCGGCATGGACGAGCTGTACAAGTCCGGCCGGACTCAGATC |
| DAF Nsig-GST-EGFP-GPI^c^ | ATGACCGTGGCTAGACCCAGCGTGCCTGCTGCCCTGCCTCTGCTGGGAGAGCTGCCTAGACTGCTGCTCCTGGTGCTCCTGTGCCTGCCTGCTGTGTGGGGA  -***GST-EGFP*** ^e^- AGCCGGACCACCAAGCACTTCCACGAGACCACCCCTAACAAGGGCAGCGGCACCACCAGCGGCACCACCCGGCTGCTGAGCGGCCACACCTGCTTCACCCTGACCGGCCTGCTGGGCACCCTGGTGACCATGGGCCTGCTGACC |
| BY55 Nsig-GST-EGFP-GPI | ATGCTGCTGGAGCCTGGCAGAGGCTGCTGCGCTCTGGCCATCCTGCTGGCCATCGTGGACATCCAGAGCGGAGGC-***GST-EGFP*** ^e^-CGGCAGCACCTGGAGTTCAGCCACAACGAGGGCACCCTGAGCAGCGGCTTCCTCCAGGAGAAGGTGTGGGTGATGCTGGTGACCAGCCTGGTGGCTCTCCAGGCCCTG |
| CEAM7 Nsig-GST-EGFP-GPI | ATGGGCAGCCCTAGCGCCTGCCCTTACAGAGTGTGCATCCCCTGGCAGGGCCTGCTGCTGACCGCCAGCCTGCTGACCTTCTGGAACCTGCCCAACAGCGCCCAGACC-***GST-EGFP*** ^e^- AGCCGGAGCGACCCTGTGACCCTGAACGTGAGATACGAGAGCGTGCAGGCCAGCAGCCCTGACCTGAGCGCCGGCACCGCCGTGAGCATCATGATCGGCGTGCTGGCTGGCATGGCTCTGATC |
| ITB3 Nsig-GST-EGFP-TM^d^ | ATGAGAGCTAGACCTAGACCTAGACCTCTGTGGGCTACCGTGCTGGCTCTGGGAGCTCTGGCTGGAGTGGGAGTGGGC-***GST-EGFP*** ^e^-ATCCTGTACGTGGTGGAGGAGCCCGAGTGCCCTAAGGGACCTGACATCCTGGTGGTGCTGCTCAGCGTGATGGGAGCTATCCTGCTGATCGGCCTGGCTGCTCTGCTGATCTGGAAGCTGCTGATCACCATCCACGCTCGGAAGGAGTTCGCCAAGTTCGAGGAAGAACGGGCTAGAGCCAAGGCCGACACCGCCAAC |
| ITAV Nsig-GST-EGFP-TM | ATGGCCTTCCCTCCTAGACGGAGACTGCGGCTGGGACCTAGAGGACTGCCTCTGCTGCTCAGCGGACTGCTCCTGCCTCTGTGCAGAGCC-***GST-EGFP*** ^e^- TGGGGAATCCAGCCTGCTCCTATGCCCGTGCCCGTGTGGGTGATCATCCTGGCCGTGCTGGCCGGCCTGCTGCTGCTGGCCGTGCTGGTGTTCGTGATGTACCGGATGGGCTTCTTCAAGGCCGTGCACCCTCCTCAGGAAGAGCAGGAACGGGAGCAGCTGCAGCCCCACGAGAACGGAGAGGGC |
| ITA5 Nsig-GST-EGFP-TM | ATGGGCAGCCGGACACCCGAGAGCCCTCTGCACGCCGTGCAGCTGAGATGGGGACCTCGGAGACGGCCTCCACTGCTGCCCCTGCTGCTGCTGCTGCTGCCACCACCTCCCAGAGTGGGAGGA-***GST-EGFP*** ^e^-GCCGTGCAGTGGACCAAGGCTGAGGGAAGCTACGGCGTGCCCCTGTGGATCATCATCCTGGCCATCCTGTTCGGCCTGCTGCTGCTGGGCCTGCTGATCTACATCCTGTACAAGCTGGGCTTCTTCAAGGCCAGCCTGCCCTACGGCACCGCTATGGAGAAGGCCCAGCTGAAGCCTCCCGCTACCAGCGACGCCTAG |

1. Glutathione S-transferase (GST)
2. Enhanced Green Fluorescent Protein (EGFP)
3. Glycosylphosphatidylinositol (GPI)
4. Transmembrane domain (TMD)
5. The sequence of Glutathione S-transferase and Enhanced Green Fluorescent Protein.

**Supplementary Table 2. Primers for sorting vector construction and gene cloing.**

| **Primer name** | **Sequence (5'-3')** |
| --- | --- |
| GST-EGFP-F | ctggttccgcgtggatccCCGGTCGCCACCATGGTG |
| EGFP-C2-DAF-R | tagtataggggagcccatCTGAGGGTGGCTCCAGGCG |
| GST-DAF-F | ATGGGCTCCCCTATACTAGG |
| GST-DAF-R | GGATCCACGCGGAACCAG |
| DAF-Nsig-NeomF | AGACAGGATGAGGATCGTTTACCATGACCGTGGCTAGACCCAGCG |
| DAF-GST-GPI-NeomR | GCGAACCCCAGAGTCCCGCCTAGGTCAGCAGGCCCATGGTCACC |
| pcDNA3.1-Neo-Up-R | AAACGATCCTCATCCTGTCT |
| pcDNA3.1-Neo-dwn-F | GCGGGACTCTGGGGTTCGC |
| CEAM7-GST-GPI-NeomF | AGACAGGATGAGGATCGTTTACCATGGGCAGCCCTAGCGCCT |
| CEAM7-GST-GPI-NeomR | GCGAACCCCAGAGTCCCGCCTAGATCAGAGCCATGCCAGCCA |
| DAF-GST-Nsig-BamF | ACCTCTCTCCCCAGGGGGATCCACCATGACCGTGGCTAGACCCA |
| DAF-GST-Nsig-BamR | TCATTGGTCTTAAAGGTACCCTAGGTCAGCAGGCCCATGGTCA |
| CEAM7-GST-Nsig-BamF | ACCTCTCTCCCCAGGGGGATCCACCATGGGCAGCCCTAGCGCCT |
| CEAM7-GST-Nsig-BamR | TCATTGGTCTTAAAGGTACCCTAGATCAGAGCCATGCCAGCCA |
| ATG10-sh-F | CCGGAGGAGTTCATGAGTGCTATAACTCGAGTTATAGCACTCATGAACTCCTTTTTTG |
| ATG10-sh-R | AATTCAAAAAAGGAGTTCATGAGTGCTATAACTCGAGTTATAGCACTCATGAACTCCT |
| ATP6AP1L-CDS-F | ataGGTACCatgagactttggaaagcaa |
| ATP6AP1L-CDS-R | atTCTAGAaacatagattttgctgatct |

**Supplementary Table 3. Primers for RT-qPCR.**

| **Primer name** | **Sequence (5'-3')** |
| --- | --- |
| actin beta-f | CAGCCATGTACGTTGCTATCCAGG |
| actin beta r | AGGTCCAGACGCAGGATGGCATG |
| GST-f | ATGGgctcccctatactagg |
| GST-r | GCCATTTATCACCTTCATCGC |
| GFP-f | ACGACGGCAACTACAAGACC |
| GFP-r | TTGTACTCCAGCTTGTGCCC |
| ATG10-f | AGACCATCAAAGGACTGTTCTGA |
| ATG10-r | GGGTAGATGCTCCTAGATGTGAC |
| ATP6AP1L-f | ACTGGCGTATATGCTCCCTCT |
| ATP6AP1L-r | CTCGACCCATCATCCGTGTC |
